# COMBINED ENDOVASCULAR AND SURGICAL THERAPY OF UTERINE FIBROMA

**Published:** 2008-02-25

**Authors:** Grigoriu Corina, Dumitrascu Mihai, Grigoras Mirela, Horhoianu Irina, Horhoianu V., Nechifor R., Dorobat B., Pavel Alina, lana G.

**Affiliations:** * Obstetrics-Gynecology Clinic - University Emergency Bucharest Hospital; ** Medical Imagistic and Radiology Clinic – Angiography and Interventional Radiology Laboratory - University Emergency Bucharest Hospital

## Abstract

Since the first description of uterine artery embolisation for the treatment of symptomatic fibroids of the uterus in 1994, this minimally invasive procedure has been increasingly performed in many countries. Transcatheter embolisation of the uterine arteries feeding large fibroids is a minimally invasive technique.

This paper presents the combined endovascular and surgical therapy in the treatment of uterine fibroma. The purpose of this therapy is saving the reproductive function of the uterus even in cases with very large fibromas or located in areas with difficult access, in which hysterectomy would have been needed. The therapy has a high rate of success, it is accompanied by disappearance of the symptoms and it has a low risk of intra- and post-operatory complications.

The first step is the embolisation of uterine arteries – a safe therapy of uterine fibroma. The procedure eliminates the risk of post-miomectomy relapse through the symultaneous devascularisation of all fibroma nodules, even of the very small ones which are unapparent clinically or imagistically.

The post-embolisation surgical intervention is undertaken in conditions of operative comfort, with minimal bleeding; it eliminates the need for blood transfusions and diminishes the duration of intervention.

Three representative cases where this therapy has been successfully applied are presented in this article.

The embolisation of the uterine arteries represents an efficient therapy of the uterine fibroma, with very good results noted in the speciality literature.

## Introduction

***The uterine fibroma and hysterectomy:***

Uterine fibroma is a very frequent benign tumor which occurs in 25% of women (female patients) in Romania. In many cases it is asymptomatic and it is discovered incidentally by a pelvic ultrasound imaging. In time, single or multiple fibromas may grow and become symptomatic by abundant, long and painful menstruations, a sensation of pressure in the abdomen, urinary frequency and back pain. These symptoms may respond initially to medical therapy (including gonadotropin-releasing hormone agonists), but often this therapy is not enough and they require surgical treatment. It often causes infertility or miscarriage. 

The standard approach of treatment of fibroid uteri includes as well surgical therapy which consists of uterus resection (hysterectomy) either transabdominal or transvaginal and sometimes resection of the ovaries. Following that intervention, the woman loses her gestational function. Furthermore, the hysterectomy has lots of risks and complications: a long recovery and social reintegration phase (6 weeks), postoperative bleeding (2%), hectic syndrome after hysterectomy (15-38), low sexual desire, depression, a high risk of cardiovascular illness. Therefore, for young women who whish to preserve their fertility, hysterectomy is not an acceptable therapeutical solution.

More recently, hormone therapy and operative endoscopy (laparoscopy and hysteroscopy) have been introduced as alternatives, together with uterine embolisation previously applied preoperatively in extensive bleeding neoplasms or to control post-partum hemorrhage.

***Segmentary myomectomy***

Segmentary myomectomy is a conservative surgical intervention which consists in the resection of tumor, the preservation the uterus and, therefore, the reproductive function. In some cases, however, the intervention is difficult or impossible because of the dimension, position or number of existing fibromas. Myomectomy is associated with increased blood loss, pain and prolonged operative time. It also has higher morbidity and mortality rates than hysterectomy. Furthermore, a significant number of patients undergoing myomectomy will need further surgical intervention. After intervention, a relapse of fibromatosis is likely to occur if there are small fibromas which have not been diagnosed clinically or imagistically before or during surgery and which continue to grow, eventually becoming symptomatic.

***Pre-surgery(preoperatively) arterial embolisation***

The embolisation of uterine arteries causes the devascularisation of all fibroma structures in the uterus, which ensures optimal conditions for surgery. The procedure consists in supraselective percutaneous catheterization of both uterine arteries followed by their embolisation with Polivinil alcohol particles (PVA) or Tachocomb fragments. The normal uterine structures are not affected, while the fibroma suffers an ischemy, necrosis and resorbtion process.

**Figure:**
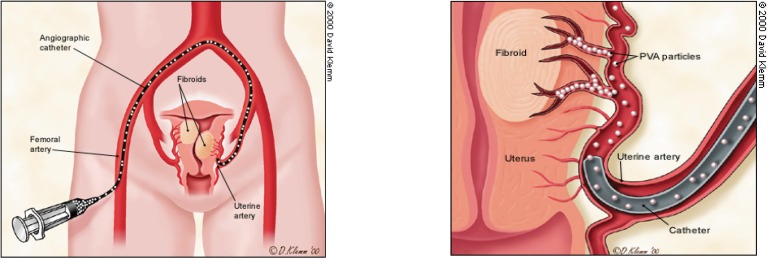


***Patient selection criteria for embolisation:***

• Young female with multiple fibromas who wish to have children

• Patients with symptomatic uterine fibroma who refuse to have a hysterectomy, blood transfusion, or general anesthesia

• Patients with uterine fibroma in pre-climax (the method is less aggressive than hysterectomy and cheaper than hormone therapy).

***The embolisation indications based on dimensions, position or number of existing fibromas:***

• Regardless of dimension, with the mention that the large embolised fibromas require good post-embolisation follow up, in order to avoid an eventually infection of the necrosed structures. Ideally, embolisation of large uterine fibroma must be followed by myomectomy.

• Regardless of number: through embolisation all the existing fibromas are devascularised simultaneously, even the smallest which can not be detected by imagistic methods.

• Regardless of localization: most indications are fibroids located intramurally and submucosally with rich vascularity. In case of pedunculated, subserosal fibromas, it is better that the embolisation be followed by myomectomy.

***The advantages of pre-surgery embolisation***

The embolisation performed a few days before surgery has numerous advantages:

- Minimal surgery bleeding;

- Eliminates the necessity for blood transfusion;

- Surgical comfort;

- Diminishes the duration of the surgery;

- Allows resection of very large fibroma or of those positioned in areas difficult to reach (the posterior side of the uterus, the inferior side close to the cervix). 

- Eliminates the risk of relapse. Through embolization, all the existing fibromas are devascularised, regardless of their dimensions and position. The fibroma structures which remain after surgery become necrotic and are resorbed during a period of several months. 

***The advantages of combined therapy***

Embolisation of uterus arteries followed by segmentary miomectomy is a conservative therapy, allowing the preservation of gestational function in cases where the usual treatment would have been hysterectomy. It is therefore, an important therapeutical option for young women, especially for those who wish to have children.

## The experience of our clinic:

At the Bucharest Universitary Emergency Hospital, through the collaboration between the Obstetrics-Gynecology Clinic and the Angiography Laboratory of the Radiology Clinic from July 2002 until present, a number of 1485 embolisations were performed. 416 of these were combined interventions (embolisation and myomectomy), all of them with positive results. Therefore, it is the only clinic in Romania where the uterine arteries embolisation is performed.

In this paper we describe three representative cases:

- Case 1: large fibroma (9cm); patient age: 20 years

- Case 2: 4cm fibroma on the posterior side of the uterus, close to the cervix, with secondary infertility; patient age: 32 years

- Case 3: uterine fibromatosis with numerous intramural and subserosal fibromas and a large nodule (10cm) situated subserosal, with infertility.

***Case 1: single large uterine fibroma***

Patient M.G. (20), from rural environment, virgo, is hospitalized with the diagnosis of pelvic-abdominal tumor.

Anamnesis: abundant and painful metroragies, urinary frequency, intraabdominal pressure.

The patient underwent: a clinical gynaecological exam, pelvic ultrasound imaging, laboratory analysis, MRI.

Diagnosis: 8/9cm single uterine intramural fibroma on the front side of the uterus.

Therapy: supraselective embolisation of blood vessels supplying blood to the fibroma, followed by segmentary myomectomy.

Embolisation of left-side uterine artery with Tachocomb fragments.

During the first day after embolisation, myomectomy and drainage surgical intervention was decided and performed. In this case, a median pubo-subombilical incision was performed. An 80/90mm fibroma was extracted with minimal bleeding.

The intraoperative histopathology exam result indicated an edematous transformed leiomyofibroma.

The postoperative evolution of the patient was favorable, without complications.

**Figure:**
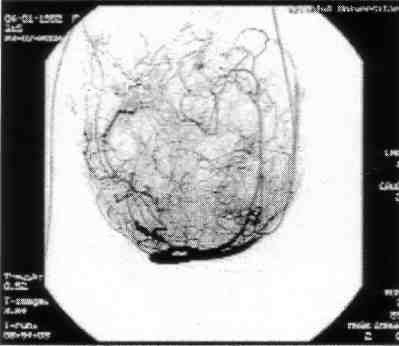
The angiography shows a large uterine fibroma (9/8cm) highly irrigated peri- and intra tumoral, connected to the left-side uterine artery

**Figure:**
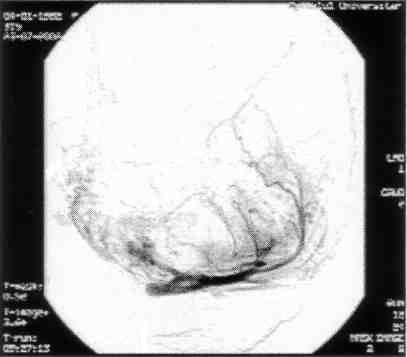
Post-embolisation result: intratumoral blood vessels are emptied, the contrast substance stagnates in the uterine artery.

**Figure:**
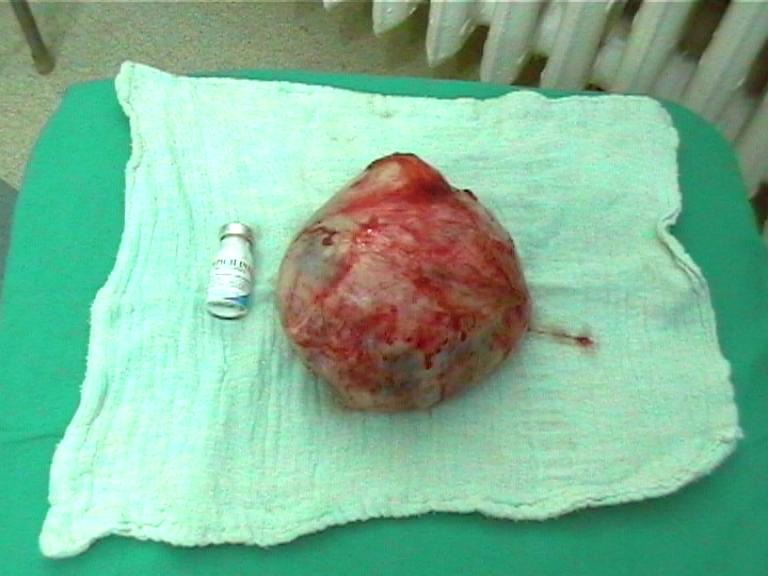
Myomectomy results: Median pubo-subombilical incision, minimum bleeding, an 80/90mm fibroma nodule is extracted. Histopathology: edematous transformed leiomyofibroma

***Case 2: single uterine fibroma, difficult position:***

Patient CL, aged 31, is hospitalised for abundant metroragy and infertility. The pelvic ultrasound imagery shows a 4/5cm fibroma, intramural, on the posterior side of the uterus, close to the cervix.

The angiographic examination shows irrigation of the fibroma. Embolisation of both uterine arteries is performed using PVA particles of 350 – 500μm.

Segmentary myomectomy is performed two days after embolisation. Under regular circumstances, the particular position of fibroma would have made its resection difficult, considering the high risk of bleeding in an inaccessible area.

Pre-surgery embolisation allows a simple and rapid resection to be performed with no bleeding. 

**Figure:**
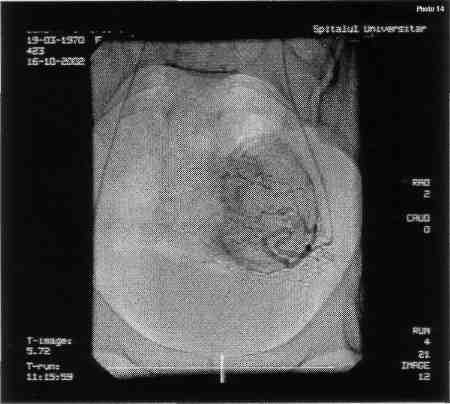
Arteriografy shows an uterine fibroma of ~4 cm, close to cervix.

**Figure:**
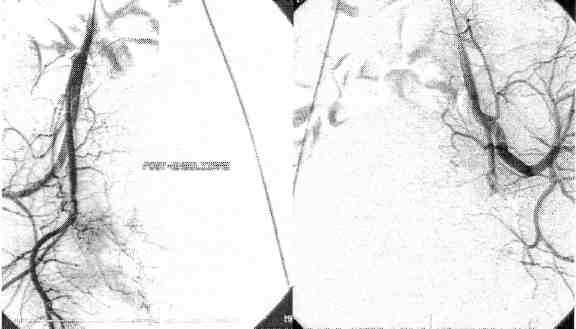
Post- embolisation result: The embolisation of both uterine arteries is performed using PVA particles of 350 – 500 microns.

**Figure:**
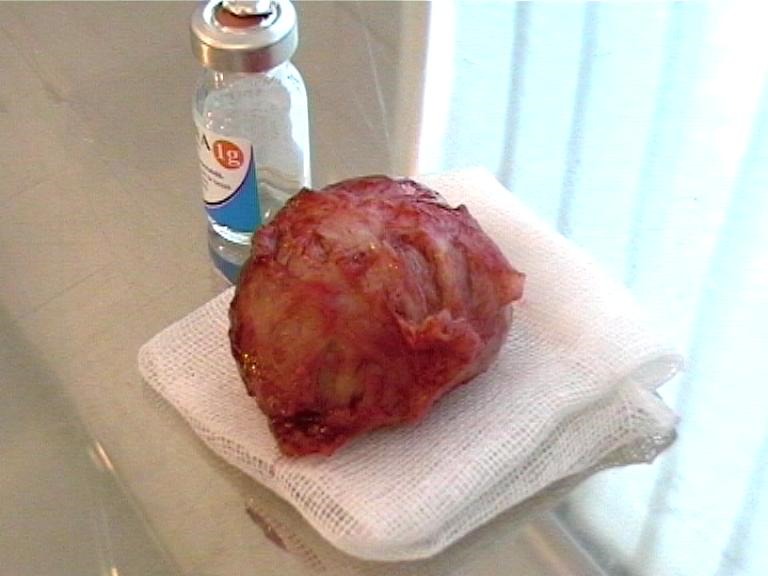
Myomectomy result: Simple and fast surgery technique, without bleeding, in an inaccessible area.

***Case 3: uterine fibromatosis***

Patient A.M., aged 36, is hospitalised for infertility. Clinical gynaecology exam and pelvic ultrasound imaging set the diagnosis of uterine fibromatosis – multiple fibroma nodules situated intramurally and subserosally, with dimensions of 2 to 5cm. The largest is a 10cm subserosal fibroma. The patient refuses hysterectomy and requests a therapeutical solution which would allow her to have children.

Embolisation of both uterine arteries with Tachocomb fragments is chosen, followed 5 days later by surgical intervention during which all fibromas and subserosal nodules were resected.

The intervention was performed in good conditions with minimal bleeding. The subserosal fibroids were eliminated. In regard to intramural tumors, embolisation of both uterine arteries led to the suppression of their vascularisation, which determined their necrosis during the following months. It is expected that in approximatively one year after embolisation, the uterus will become fertile again.

**Figure:**
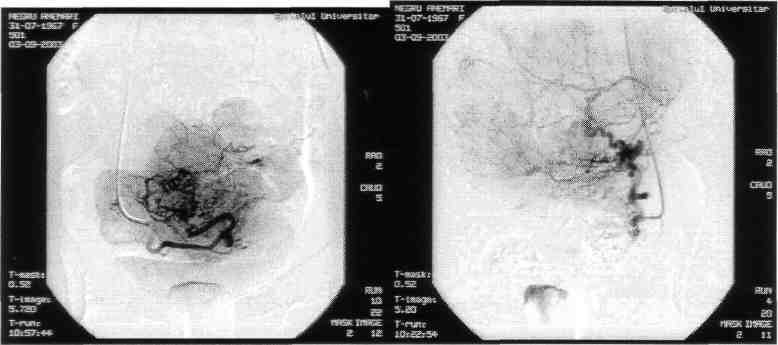
Angiography shows: Multiple uterine fibroids with a rich vascularisation, situated both intramurally and subserosally. Both uterine arteries participate at the uterine fibroids irrigation.

**Figure:**
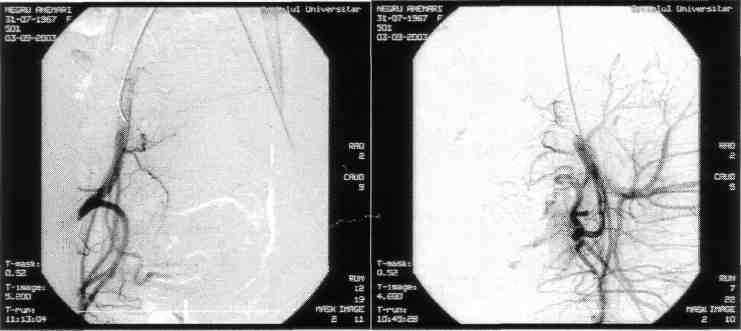
Post-embolisation result: Favorable post-embolisation result with suppression of their vascularisation, the contrast substance stagnates in both uterine arteries.

**Figure:**
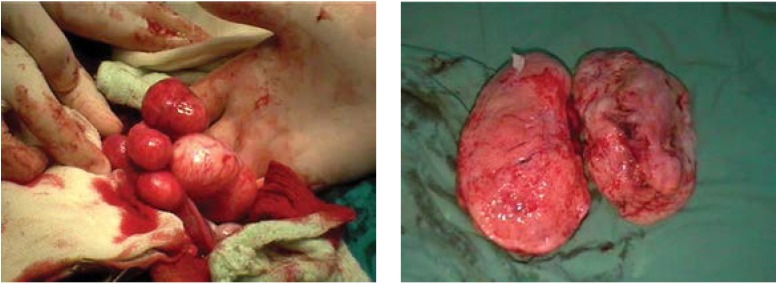
Myomectomy result: Multiple uterine fibroids situated subserosally with various dimensions and a pedunculated fibroma of ~ 10cm

## Conclusions:

Embolisation of the uterine arteries is a safe and efficient method of therapy for uterine fibromas. This procedure eliminates the risk of post-myomectomy relapse through simultaneous devascularisation of all uterine fibroids, even very small ones which are unapparent clinically or imagistically.

It provides conditions for a conservative surgical intervention which keeps the reproductive function of the uterus, even in cases with very large fibromas for which otherwise hysterectomy would have been necessary.

Post-embolisation surgical intervention is undertaken in conditions of operative comfort, with minimal bleeding; it eliminates the need for blood transfusions and diminishes the duration of intervention.

It is the best option in cases where surgical intervention is not recommended or refused by the patient, obviously when there are no restrictions for using this therapy.
